# Eye Movement Evidence for Simultaneous Cognitive Processing in Reading

**DOI:** 10.3390/children10121855

**Published:** 2023-11-26

**Authors:** Argyro Fella, Maria Loizou, Christoforos Christoforou, Timothy C. Papadopoulos

**Affiliations:** 1 School of Education, University of Nicosia, Nicosia 1700, Cyprus; fella.a@unic.ac.cy; 2 Ministry of Education, Sport, and Youth, Nicosia 1434, Cyprus; moizoup@te.schools.ac.cy; 3 Division of Computer Science, Mathematics and Science, St. John’s University, New York, NY 11439, USA; christoc@stjohns.edu; 4 Department of Psychology, Center for Applied Neuroscience, University of Cyprus, Nicosia 1678, Cyprus

**Keywords:** simultaneous processing, eye movements, reading difficulties, reading-level match design, consistent orthographies

## Abstract

Measuring simultaneous processing, a reliable predictor of reading development and reading difficulties (RDs), has traditionally involved cognitive tasks that test reaction or response time, which only capture the efficiency at the output processing stage and neglect the internal stages of information processing. However, with eye-tracking methodology, we can reveal the underlying temporal and spatial processes involved in simultaneous processing and investigate whether these processes are equivalent across chronological or reading age groups. This study used eye-tracking to investigate the simultaneous processing abilities of 15 Grade 6 and 15 Grade 3 children with RDs and their chronological-age controls (15 in each Grade). The Grade 3 typical readers were used as reading-level (RL) controls for the Grade 6 RD group. Participants were required to listen to a question and then point to a picture among four competing illustrations demonstrating the spatial relationship raised in the question. Two eye movements (fixations and saccades) were recorded using the EyeLink 1000 Plus eye-tracking system. The results showed that the Grade 3 RD group produced more and longer fixations than their CA controls, indicating that the pattern of eye movements of young children with RD is typically deficient compared to that of their typically developing counterparts when processing verbal and spatial stimuli simultaneously. However, no differences were observed between the Grade 6 groups in eye movement measures. Notably, the Grade 6 RD group outperformed the RL-matched Grade 3 group, yielding significantly fewer and shorter fixations. The discussion centers on the role of the eye-tracking method as a reliable means of deciphering the simultaneous cognitive processing involved in learning.

## 1. Introduction

Simultaneous cognitive processing, which refers to the handling and making sense of multiple pieces of information, such as images and verbal stimuli, is crucial for children’s cognitive growth and learning (see [1,2,3,4] for reviews). Likewise, the detrimental effects of poor simultaneous processing on academic achievement have been documented in various learning subjects (e.g., reading, writing, math, or physical activities) (e.g., [5,6,7,8]) and developmental groups (e.g., [9,10,11,12]). However, research exploring the role of simultaneous cognitive processing in learning employs primarily cognitive measures (e.g., [13,14]). Studies have shown that eye tracking provides a more comprehensive and dynamic perspective on information processing than traditional cognitive behavioral measures, which only offer accuracy and response time at a single time point at the output stage (e.g., [15]). Eye tracking captures detailed information about allocating visual attention and cognitive resources throughout a task (e.g., [16]), allowing researchers to investigate the temporal and spatial aspects of cognitive processes and uncover the efficiency and effectiveness of information processing. However, no research has yet explored whether eye movements during simultaneous processing can distinguish between children with learning disorders and those without.

### 1.1. Simultaneous Processing, Reading, and Learning: Behavioral and Cognitive Research

Over the past 30 years, systematic behavioral research in reading has led to the development of several theories about how reading could be best acquired and remedied, particularly in the context of reading difficulties. The research has shown that reading is a complex process that requires concurrently using a wide range of cognitive and linguistic skills (e.g., [17,18,19,20]). One of the reading-related skills that has received particular attention is simultaneous processing (e.g., [1,2,6]). Simultaneous processing is a type of information processing [21] used in the PASS (Planning, Attention, Simultaneous, and Successive Processing) theory of intelligence (e.g., [3,22]). It has been suggested that this type of processing is used in skills such as word recognition and sentence or text comprehension (e.g., [13,23,24]). Several studies have shown that simultaneous processing plays a critical *direct* role in reading development across languages with transparent orthography (e.g., [25]), such as Greek, German, or Finnish, where the relationship between graphemes and phonemes is straightforward. It is also essential in nontransparent orthographies (e.g., [6,26,27]), like English, French, or Danish, where the mapping between orthography and phonology is inconsistent; see [28]. A recent meta-analytic study [6] reported a moderate-to-strong correlation between simultaneous processing and reading performance (r = 0.36 for accuracy, r = 0.31 for fluency, and r = 0.42 for comprehension). Keat and Ismail [29] revealed an equally strong correlation (r = 0.72). According to dual-route theories of word recognition, simultaneous processing is commonly used when readers identify each word as an orthographic unit (see [3,7] for discussion). This occurs when a word is recognized visually rather than through the sounds of its letters.

Also, several studies suggest that reading development benefits from simultaneous processing. This is believed to occur *indirectly*, through the effects of central linguistic skills, such as phonological awareness (i.e., the ability to perceive, analyze and manipulate the sound units in spoken languages [30]), RAN (i.e., the ability to name visually presented stimuli such as digits and letters as fast as possible [31]) or orthographic processing (i.e., the “memory for specific visual/spelling patterns” [32] (p. 47), and “the rapid recognition of sight words” [33] (p. 73) (e.g., [7,13,25]). For example, Papadopoulos et al. [25] followed a group of Greek-speaking readers from Grade 1 to Grade 2 and found that in Grade 1, simultaneous processing predicted literacy skills (reading and spelling) through RAN and phonological awareness. In Grade 2, simultaneous processing predicted literacy skills directly. These findings indicated that manipulating spoken language sounds and rapidly naming a series of letters requires the indirect support of more general and modality-unspecific processes, such as simultaneous processing (Grade 1). As children become more experienced with reading, they learn recurring letter patterns, which results in a direct relationship between simultaneous processing and literacy skills (Grade 2). Notably, this relationship between simultaneous processing and reading skills is consistent across ages, from childhood to adulthood (e.g., [7,27,34]). Papadopoulos et al. [34] also found that cognitive and linguistic processes indirectly predict excellence in reading achievement before formal reading instruction begins. Similarly, Georgiou and Das [27] reported that simultaneous processing predicts reading comprehension both directly and through the effects of text-reading fluency in a study with university students.

Similar findings are reported for math (e.g., [26,35]), writing (e.g., [36,37]), spelling (e.g., [25]), or physical activities (e.g., [10,38,39]). Since the present study focuses on the relationship between simultaneous processing and reading, we do not detail the findings in other developmental areas. However, it is worth noting that a strong foundation in simultaneous processing is essential for performing motor tasks and developing math and writing skills.

Furthermore, individuals with reading difficulties often struggle with integrating stimuli and understanding task components due to simultaneous processing deficits (see [3,4]). For instance, Keat and Ismail [40] found that children with RDs had significantly lower performance in tasks that affected their simultaneous and successive processing, attention, and planning processes. Combining simultaneous processing and other cognitive (PASS) processes could help diagnose RDs [41].

In addition, deficits in simultaneous processing tasks have been observed across different languages (e.g., [12,42,43]) and developmental levels from childhood to adulthood (e.g., [14,44,45]). For example, Elwan et al. [44] studied the cognitive profiles of 180 Egyptian children with and without RDs across three age groups: middle childhood age (7–9 years old), late childhood stage (9–12.5 years old), and early adolescence (12.5–15 years old). Results indicated that children with reading difficulties performed significantly worse than their age-matched peers in tasks requiring simultaneous processing. Some researchers attribute these difficulties to deficits in the number of distinct visual elements that can be processed simultaneously, considering the importance of visual attention capacity in reading (e.g., [46,47]).

### 1.2. Reading Difficulties and the Reading-Level-Matched Design

Whether processing skills, like linguistic skills, cause or result from reading difficulties is a contentious issue. Bryant and Goswami [48] had long suggested that processing skills, significantly worse in the RD group than in younger readers matched on reading ability, play a causal role in RDs. Several researchers tested this hypothesis by comparing the processing skill profiles of younger typical and older RD groups, carefully matched on reading ability [49,50,51]. This evidence suggests that if reading development is responsible for developing a reading-related processing skill such as phonological awareness and RAN, older participants with RD should perform worse in that processing skill compared to younger typical readers with the same reading level. In one of these studies, Swan and Goswami [52] found that children with RD performed significantly worse than their RL-matched controls on phonological awareness tasks. These findings suggest that a specific deficit, such as a phonological deficit, may be causally related to reading difficulties or that an associated lag in developing reading-related skills causes reading problems [53].

Consequently, the RL-match design has been favored for comparing groups in studies examining various reading-related skills that may contribute to reading difficulties (e.g., [49]). This method has been commonly used to explore the causal role of linguistic factors, including phonological awareness (e.g., [54]), RAN (e.g., [55,56]), or orthographic processing (e.g., [57]) in reading difficulties. The effectiveness of the RL-match design has been tested in alphabetic writing systems (both inconsistent [58,59] and consistent orthographies [51,60]) and non-alphabetic languages, such as Chinese (e.g., [61,62]).

The research shows that children with reading difficulties learning to read in English, a language with an inconsistent orthography, perform poorer on phonological awareness and RAN tasks than chronological-age children and reading level-matched (e.g., [58,63]). For example, Katzir et al. [64] conducted a study that compared English-speaking children with RDs to RL-matched controls on reading-related tasks, such as phonological awareness, RAN, and orthographic processing. The results showed that children with RD had difficulties in phonological awareness, RAN, and orthographic processing compared to their RL-matched controls. This suggests that the above skills could be considered potential causes of reading disorders in inconsistent orthographies (e.g., [48]).

In contrast, research in consistent orthographies, such as Italian and Greek, has yielded controversial results (e.g., [50,56]). For example, Tobia and Marzocchi [51] reported that Italian-speaking children with RD were significantly slower in rapid naming than CA and RL-matched controls. However, Georgiou et al. [56] found that Greek-speaking children with RD performed similarly to RL-matched controls in reading-related tasks such as phonological awareness and rapid naming. This suggests that linguistic difficulties in consistent orthographies may result from delayed reading development regardless of how well reading-related linguistic factors develop.

It is worth noting that few behavioral studies have used a reading-level match design to explore the potential relationship between simultaneous processing and reading difficulties. For example, Wang et al. [43] conducted a study on 27 Grade 4 children with RDs and 27 CA-matched and 27 Grade 2 RL-matched controls to evaluate their performance on various reading-related skills, such as simultaneous processing, successive processing, attention, planning, phonological awareness, and RAN. They found that Chinese-speaking children with RD had deficits in simultaneous processing compared to CA-matched controls, but they performed similarly to RL-matched controls. Likewise, Papadopoulos and Kendeou [24] found that the Grade 1 Greek-speaking children with RDs performed as well as the RL-matched group in simultaneous processing measures, which led them to conclude that simultaneous processing skills are not causally related to reading difficulties.

More research is needed to determine whether the reading-level matched design is appropriate for studying reading difficulties when focusing on reading-related skills such as simultaneous processing. This may require more advanced methods to validate conclusions from behavioral studies and further understand the role of simultaneous processing in reading and related difficulties.

### 1.3. Simultaneous Processing and Reading: Eye-Tracking Research

Eye tracking is a methodology developed to understand the nature of reading difficulties better. This technique captures a person’s eye movements across a screen as readers interact with text and images (e.g., [65,66]). Unlike conventional metrics that only measure efficiency and effectiveness at the output processing stage (e.g., [15]), eye-tracking metrics can provide significant insights into readers’ cognitive processes [67]. With its high temporal resolution of milliseconds (e.g., [68]), eye-tracking methodology can reveal the internal cognitive stages at which information processing occurs (e.g., [69]). For instance, eye movements’ characteristics, such as duration of fixations, may reflect the efficiency of visual/orthographic acquisition from the target stimulus (e.g., [70]), the size of attentional focus (e.g., [71]) or the degree of automaticity in accessing phonological or visual representations from items array (e.g., [16]). However, it is not yet fully understood whether eye movements during reading-related tasks reflect reading difficulties.

Eye-tracking research has shown that individuals with reading difficulties have different eye movement patterns than typically developing readers of the same age (e.g., [70,71,72]). Specifically, they tend to make more and longer fixations, more and shorter saccades, and more regressions. These differences in eye movements have been observed in various cognitive and linguistic tasks, such as RAN (e.g., [70,73,74]) and orthographic processing (e.g., [57]). As a result, individuals with RD have reduced efficiency in extracting information when processing reading-related tasks [70,71].

However, only some eye-tracking studies have used a reading-level match design to investigate causality assumptions [16,70,71]. In such studies, children with RDs produced eye movement patterns similar to their RL-matched controls in reading-related tasks, such as RAN. For example, Peters et al. [71] found that children with RDs (aged 7–9) produced more and longer fixations in the RAN tasks when compared to their CA-matched controls but not when compared to their RL-matched controls. The authors suggested that children with RDs require similar attentional resources and the same amount of time for necessary cognitive processes, such as magnocellular processing, during RAN tasks as their RL-matched controls. Similarly, Fella et al. [16] found that children with RDs produced more and longer fixations and regressions and more saccades in the RAN tasks when compared to their CA-matched controls but not when compared to their RL-matched controls. The authors concluded that deficits in rapid naming skills are a consequence, rather than a cause, of reading failure. Therefore, eye-tracking research offers a promising avenue to investigate whether the reading-level matched design is appropriate for testing causal theories of reading difficulties beyond the evidence available from behavioral data.

While the eye movement method has contributed to researching the linguistic and cognitive factors that differentiate children with RD and their controls (CA and RL), the relevant studies have at least three significant limitations. First, previous eye-tracking research has focused on studying processes or skills like RAN (e.g., [70,73]) or orthographic processing (e.g., [57]), while fundamental cognitive abilities, like simultaneous processing, have yet to be noticed. Second, the studies examining how eye movements during reading-related tasks can distinguish between children with RDs and those without have typically included participants whose first language was English (e.g., [72,73]), leading to limited evidence of eye measures’ effectiveness in languages with consistent orthographies. Finally, the reading-level match design’s appropriateness for studying reading-related skills using eye-tracking methods remains unclear, and there has been no systematic investigation into possible differences in eye movements between children with reading difficulties (RDs) and their age- and reading-matched controls concerning their performance on simultaneous processing tasks.

The current study aimed to overcome previous limitations by using eye-tracking technology to examine the cognitive resources involved in simultaneous processing. Specifically, we sought to determine if these processes are the same across groups of children with different reading abilities. To achieve this goal, we compared eye movement patterns (fixations and saccades) between Greek-speaking children with RDs and control groups (children of chronological age and reading level) during a simultaneous processing task. Based on previous research that has used simultaneous processing tasks to differentiate between children with RDs and their CA controls (e.g., [3]), we hypothesized that children with reading difficulties would exhibit more and longer fixations than their age-matched controls. These differences would suggest greater difficulty processing verbal and spatial information presented simultaneously in children with RDs. Additionally, we hypothesized that the older children with RDs would not differ in eye movement patterns from younger RL-matched controls, indicating that simultaneous processing skills are not necessarily a defining cause of reading difficulties in a consistent orthography (e.g., [24]).

## 2. Materials and Methods

### 2.1. Participants

Sixty children (36 males, 24 females; age range = 7.6 through 12.1 years) from 15 typical urban and rural schools in Cyprus participated in the study. Schools were randomly chosen from those traditionally collaborating with the Department of Psychology, University of Cyprus, for research purposes. All schools followed the same reading curriculum the Ministry of Education, Sport and Youth provided. All children were native Greek speakers with no reported history of cognitive, attentional, sensory, or behavioral difficulties. Children receiving speech and language therapy services were excluded from the sample to ensure that reading deficits were not confounded with speech problems. The children were recruited from schools of an average socioeconomic range based on the schools’ location. The schools did not provide information on parents’ educational level and profession. Based on the stepwise group selection process described below, the sample was divided into four groups: the Grade 3 and Grade 6 children with RDs and their CA controls (Table 1).

Step I for group selection: When selecting participants for the study, we asked teachers to nominate children from their classrooms who had difficulty decoding words at an age-appropriate rate in the Greek language (e.g., [16]) but had no sensory, intellectual, or attention-related problems. Research has shown that teachers’ judgments about their students’ reading levels are generally confirmed by the children’s subsequent reading scores (e.g., [75]). Therefore, we asked teachers to rate the nominated children independently by completing a 12-item reading-ability checklist. Items were scored on a Likert-type scale ranging from 1 (does not apply) to 4 (definitely applies). The children with teachers’ ratings less than the 20th percentile on the reading ability scale were selected for the RD groups. Cronbach’s alpha for this scale is reported to be 0.90 (see [76]).

Step II for group selection: Once parental consent was obtained, the selected children were tested on reading fluency and general cognitive ability measures to ensure they met the inclusionary criteria for reading difficulties, as described in the Diagnostic and Statistical Manual of Mental Disorders [77]. We used reading fluency tasks as dependent measures to evaluate the reading rate despite the complexity of the words, as children with RDs in Greek tend to perform well in accuracy (e.g., [13]). A similar cut-off score was used in previous studies with Greek-speaking participants (e.g., [16,56]). It is important to note that Greek is a transparent language with few inconsistencies at the grapheme-phoneme level [78]. Fifteen Grade 3 (8 males, 7 females; mean age = 8.30 years, SD = 0.27) and 15 Grade 6 children (11 males, 4 females; mean age = 11.01 years, SD = 0.92) were included in the study. All of these children had scored at least one standard deviation below their respective age-group mean on the reading fluency tasks (word reading fluency and phonemic decoding fluency; ERS-AB; [79]) but had scored within the average range on verbal (assessed using [80]; Greek standardization [81]) and non-verbal ability tasks (assessed using Nonverbal Matrices from the DN:CAS, [82]; Greek standardization [83]). The participants in both experimental groups had not been formally diagnosed with any specific learning difficulties related to reading and writing.

Step III for group selection: Another group of 15 Grade 3 (9 males, 6 females; mean age = 8.36 years, SD = 0.42) and a group of 15 Grade 6 children (8 males, 7 females; mean age = 11.47, SD = 0.32) with age-appropriate reading fluency skills were randomly chosen from the same classes and were matched to the RD groups based on chronological age and gender. The Grade 3 group was used as a control RL-matched group for the Grade 6 RD group. MANOVA analysis showed that the RD groups performed significantly worse than their CA-matched controls in reading fluency tasks. However, no differences were observed between the Grade 6 RD group and their RL-matched controls in the reading tasks. Moreover, to ensure that reading deficits were not due to verbal and non-verbal ability deficits, a MANOVA analysis with a set of control measures was performed. Results showed no differences between RD and control groups for verbal and non-verbal ability measures. The characteristics of the participants are summarized in Table 2.

### 2.2. Behavioral Measures

#### 2.2.1. Reading Fluency

The participants’ word reading ability was evaluated using two tasks from the standardized Early Reading Skills Assessment Battery [79]: a word reading fluency and a phonemic decoding fluency task. In each task, the participants were instructed to accurately and quickly read the list of words or nonwords within a minute. The fluency score of each participant (which represented the number of words or nonwords read correctly within 60 s in each task) was recorded. The real word and nonword lists were preceded by a practice list to familiarize participants with the task requirements.

- Word reading fluency (WRF). The word list in this task comprised 80 words forming a 2 × 2 × 2 factorial design [frequency (high/low), orthographic regularity (regular/irregular; e.g., /τόπι/;/topi/; ball vs. /έννοια/;/ennia/; concept), and length (bisyllable/trisyllable)]. The list consisted of nouns, with a few adjectives and verbs. Cronbach’s alpha for this task is reported to be 0.92 in Grades 3 and 6 [84].
- Phonemic decoding fluency (PDF). The word list in this task comprised 45 pronounceable nonwords. These nonwords were created by altering two or three letters from real words, either by substituting them or by using them backwards, e.g., /σχολείο/;/sxoleo/; school). The task began with one-syllable words and progressed to five-syllable words. Cronbach’s alpha for this task is 0.89 in Grades 3 and 6 [84].

#### 2.2.2. Verbal Ability

The participants’ verbal ability was evaluated using the Vocabulary Subtest from the Wechsler Intelligence Scale for Children-III (WISC-III-R [80]; Greek Adaption [81]). The examiner presented 30 words orally, and the participants were asked to provide verbal definitions for each word. The experimenter assigned a score of 0, 1, or 2 based on the understanding and richness of expression for each answer. If the participants provided four consecutive incorrect answers, the subtest was ended. For Grades 3 and 6, the Cronbach’s alpha reliability coefficient is 0.81 [81].

#### 2.2.3. Non-Verbal Ability

Non-verbal ability was evaluated using the Matrices subtest from the DN-Cognitive Assessment System (DN:CAS, [82]; Greek standardization [83]). This 33-item multiple-choice test requires participants to identify patterns and relationships between geometric shapes. Participants had to decode the relationship between the item parts and select the best option. The test was discontinued after four consecutive incorrect answers. The total number of correct responses was the participants’ score. Papadopoulos et al. [83] report that Cronbach’s alpha reliability coefficients for Grades 3 and 6 are 0.73 and 0.78, respectively.

### 2.3. Eye-Tracking Measures

**Figure 1:**
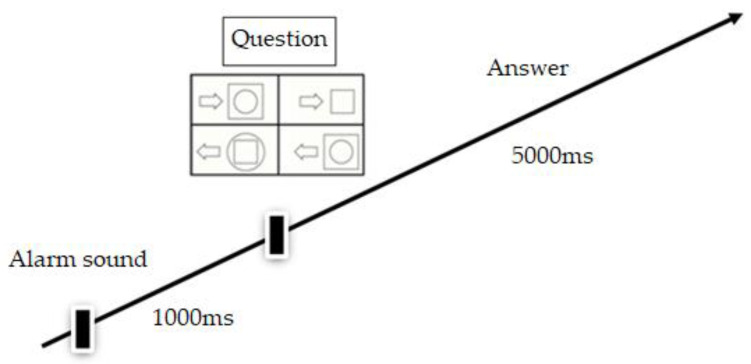
Sample stimuli of the VSR task.

Simultaneous Processing Task. Simultaneous processing was assessed with the Verbal–Spatial Relations (VSR) task (DN:CAS, [82]; Greek standardization [83]). A computerized version of the VSR task was adapted from the work of Okuhata et al. [85] and administered through an eye tracker (see next Section). It required participants to listen to a question and then point to a picture among four competing illustrations demonstrating the spatial relationship raised in the question (Figure 1). For example, the item “Which picture shows the ball in the basket on the table” included four pictures, three distractor pictures and one target picture matching the description. Cronbach’s alpha reliability coefficient was 0.72 for Grades 3 and 6. The VSR task consisted of 27 items in ascending order of difficulty. Two scores were recorded: the total number of items answered correctly (accuracy) and the time taken to complete the task (latency). Furthermore, four interest areas were created for each item: one for the target picture and three for the distractor pictures, providing output for the eye-movement data related to each interest area.

### 2.4. Procedure

Participants underwent individual testing that lasted for approximately 40 min. The tasks’ administration order remained constant for all the participants. The reading fluency, verbal, and non-verbal ability tasks were administered first, followed by the simultaneous processing measure, which involved using the eye tracker. After completing half of the testing, all participants were given a 5-min break to control for likely fatigue. The testing was conducted in a testing room in the Center for Applied Neuroscience, University of Cyprus, during extracurricular hours, such as weekends. Written permission from schools and parents was obtained before testing. The Cyprus National Bioethics Committee approved the study.

### 2.5. Eye Tracking and Data Manipulation

We used the EyeLink 1000 Plus eye-tracking system (SR Research, Kanata, ON, Canada) to record eye movement data. The items were displayed on an ASUS VG-236 monitor (1920 × 1080, 120 Hz, 52 × 29 cm) connected to a Dell Precision T5500 workstation. All participants were seated comfortably at 60 cm from the monitor. We aimed to collect data using methods consistent with the standards of the field and the guidelines provided by the EyeLink 1000 Plus system’s manufacturer. Therefore, we opted for a monocular recording approach to achieve highly accurate and head-stabilized recording. To achieve this, we used a camera mounted on a Desktop Mount and a chinrest to enable monocular data acquisition. Eye position was calibrated based on the right eye using nine random fixation points, and all recordings and calibrations were carried out monocularly while viewing was binocular (see also [16,70]). The sampling rate was 1000 Hz, and the calibration process was repeated to verify calibration accuracy.

We used the EyeLink Data Viewer [86] to visualize and process the recorded eye movement data (for further information, see [16]). We extracted twelve variables of interest, which included the number and duration of fixations, the number and duration of fixations on the target picture, the number and duration of fixations on the distractor pictures, the number and duration of saccades, the number and duration of saccades on the target picture, and the number and duration of saccades on the distractor pictures. These variables were averaged to obtain the average number and duration of eye movements for each correct response (see [57]).

### 2.6. Data Analysis

Initially, we aimed to investigate the deficits in the behavioral simultaneous processing task. We conducted a MANOVA analysis with accuracy and time in the SVR task as the dependent variables to achieve this objective. In addition, we analyzed the eye movement measures of children with RDs compared to their CA and RL-matched controls in the simultaneous processing task. We examined the number and duration of fixations and saccades on the target and distractor pictures in four separate MANOVA analyses with the groups (4) as a fixed factor. These analyses aimed to uncover the cognitive resources involved in the simultaneous processing of children of different ages and reading levels. To determine the effect size for differences between group means, we used Eta squared (*η*^2^) and Cohen’s *d* (a standardized difference between two means, with a generally accepted minimum level of power of 0.80, [87]).

## 3. Results

### 3.1. Simultaneous Processing Task: Behavioral Data

MANOVA analysis was performed, with descriptive variables (accuracy and time) in the SVR task as the dependent measures and groups (4) as a fixed factor. The main group effects were significant; Wilks’ L = 0.645, F(6,110) = 4.50, *p* < 0.001, η^2^ = 0.20. Subsequent univariate analyses showed that the main effect of the group was significant for all measures (Table 3). Additionally, effect size comparisons (Cohen’s d) showed significant differences between children with RDs and control groups. The Grade 3 RD group was significantly less accurate (d = 1.13) than the CA-matched group in the SVR task. In addition, the Grade 6 RD group outperformed the RL-matched Grade 3 group, spending significantly less time processing the SVR task (d = 1.00). Finally, no significant differences were observed between the Grade 6 groups in the SVR task (Table 3).

### 3.2. Simultaneous Processing Task: Eye Movement Data

A MANOVA was performed with groups (4) as a fixed factor, and the fixations count measures (total number of fixations, number of fixations on the target picture, and number of fixations on the distractor pictures) as dependent measures. The main effect of group was significant; Wilks’ L = 0.715, F(9,131) = 2.16, *p* < 0.05, η^2^ = 0.11. Subsequent univariate analyses showed that the main effect of the group was significant in all instances: F(3,56) = 5.15, *p* < 0.01, η^2^ = 0.22 for the total number of fixations; F(3,56) = 4.36, *p* < 0.01, η^2^ = 0.19 for the number of fixations on the target picture; and F(3,56) = 6.30, *p* = 0.001, η^2^ = 0.25 for the number of fixations on the distractor pictures. Effect size comparisons showed significant differences between the Grade 3 reading ability groups. Specifically, the Grade 3 RD group performed significantly more fixations than their CA-matched controls in the total number of fixations (d = 0.93) and the number of fixations on the distractor pictures (d = 1.07). Furthermore, the Grade 6 RD group outperformed the RL-matched Grade 3 group, yielding significantly fewer fixations on the distractor areas of interest (d = 0.75). Finally, no significant differences were observed between the Grade 6 groups in the fixation count measurements (Table 4).

Similarly, a MANOVA with the fixation duration measurements (total fixation duration, fixation duration on the target picture, and fixation duration on the distractor pictures) as the dependent measures indicated a significant main effect of group; Wilks’ L = 0.649, F(9,131) = 2.84, *p* < 0.01, η^2^ = 0.13. Subsequent univariate analyses showed that the main effect of the group was significant for all measures: F(3,56) = 4.91, *p* < 0.01, η^2^ = 0.21 for the duration of the total fixations; F(3,56) = 4.94, *p* < 0.01, η^2^ = 0.21 for the duration of the fixations on the target picture; and F(3,56) = 8.27, *p* < 0.001, η^2^ = 0.31 for the duration of the fixations on the distractor pictures (Table 4). Effect size comparisons revealed that the Grade 3 RD group produced significantly longer fixations than the CA controls in the areas of interest containing the distractor items (d = 0.73). Furthermore, the Grade 6 RD group performed shorter fixations than the RL-matched Grade 3 group in total (d = 0.94) and the distractor areas of interest (d = 1.07). Finally, no significant differences between the Grade 6 groups in the fixation duration measures were observed.

Finally, the number (total number of saccades, number of saccades on the target picture, and number of saccades on the distractor pictures) and the duration of saccades measures (total saccade duration, saccade duration on the target picture, and saccade duration on the distractor pictures) were examined in a separate MANOVA with the groups (4) as a fixed factor. The main effect of the group was not significant; Wilks’ L = 0.787, F(9, 131) = 1.51, *p* = 0.149 for the number of saccades; and Wilks’ L = 0.859, F(9, 131) = 0.64, *p* = 0.491 for the saccade duration.

## 4. Discussion

This study examined the cognitive resources involved in simultaneous processing and whether they are the same across chronological or reading age groups. Specifically, we looked at eye movements (fixations and saccades) of Greek-speaking children with RD and compared them to controls (CA and RL) during a simultaneous processing task. This study is important because previous research focused mainly on the contribution of central linguistic skills, such as phonological awareness, 88 and 89, and RAN: 15, 16, 70, to reading development and related difficulties while neglecting the significant role of cognitive skills in manifesting reading difficulties. Furthermore, we applied a reading-level match design for the first time to examine the causal relationship between simultaneous processing and reading using eye-tracking measurements. We tested two hypotheses: first, that children in the RD groups (Grades 3 and 6) would show impairments in eye movement compared to their CA-matched controls, and second, that the older children with RDs would not exhibit a deficit in eye movement measures, as they were carefully matched with the RL-matched controls on tasks relevant to a dyslexia diagnosis (see [50] for a pertinent argument). The results confirmed the second hypothesis and partly the first one, demonstrating that only the younger age group had deficient eye movements when processing verbal and spatial stimuli simultaneously, compared to their typically developing counterparts (see [3]). It is worth noting that the Grade 6 RD group performed similarly to the RL-matched group in saccade measures and better than the RL-matched group in fixation measures [16,57].

Our findings have significantly contributed to the existing literature in four aspects. First, group differences in the number and duration of fixations among the Grade 3 groups demonstrate that verbal and spatial relation processing can explain group differences in reading performance (e.g., [3,40]), especially in the early stages of reading (e.g., [13,17]). Indeed, fixation measures reflect cognitive processing and the ability to process multiple stimuli simultaneously (e.g., [88,89,90,91,92,93]). Our findings indicate that Grade 3 children with RDs require more time to simultaneously process verbal and spatial stimuli than their typically developing counterparts [see also 16 for similar conclusions]. This processing difficulty arises when integrating stimuli and understanding task components (e.g., [94]), particularly when distracting features surround the target stimuli (e.g., [95,96]).

As a result, Grade 3 poor readers had difficulty efficiently processing information in distractor areas. They tended to fixate on the distractor pictures for extended periods, examining each element closely. This type of processing resulted in increased fixation duration and frequency in areas with distractor items of interest. Previous eye-movement studies have consistently supported that individuals with RDs process visual stimuli one at a time and do not take as much advantage of parafoveal processing as good readers, which can negatively impact their reading speed and ability (e.g., [97,98]). Our data also confirmed that Grade 3 typical readers integrated separate stimuli into a single whole (see [99]), resulting in shorter times, especially in distractor areas. Thus, the reduced parafoveal processing in individuals with RDs highlights one of the challenges they face in developing proficient reading skills and has implications for designing effective interventions and strategies to improve their reading abilities. Tailored approaches, such as digital tools and assistive technology applications, are designed to provide simultaneous visual and verbal support for reading (e.g., [100,101]).

Second, our results showed that the Grade 6 RD children performed similarly to the control group in the simultaneous processing task (e.g., [27,102]). This finding suggests at least two things. First, students in Grade 6 with RDs have developed comparable simultaneous processing skills and use strategies similar to those of their typically developing peers. Moreover, their reading ability does not impact this performance (see [13]). Second, the influence of simultaneous processing skills on reading performance may not be direct in the upper elementary school years (e.g., [5,7]). As children gain reading experience, distal cognitive skills (more general and modality-unspecific), like simultaneous processing, may indirectly affect reading difficulties (e.g., [5]). Distal cognitive skills indirectly affect reading via proximal, primarily linguistic skills, such as phonological awareness, rapid naming, or orthographic processing (e.g., [25,34]). It has been long shown that phonological awareness and orthographic processing are the most reliable and consistent proximal predictors of reading development [13,17]. Therefore, future research should consider using eye movement measures during cognitive and linguistic tasks to understand the nature of reading difficulties and the role of distal and proximal processes in reading.

Third, our study found no significant differences in saccadic measures between children with and without reading difficulties (RDs). This is consistent with previous research on non-reading tasks (e.g., [103]). Our results suggest that RD and control groups use a similar strategy to parse the visual stimuli, such as target and distractor pictures, by breaking them down into smaller sub-units (e.g., [57]). Previous studies have shown that when engaging in complex search tasks, participants focus first on the center of the visual stimuli and then on the centers of successively smaller groupings of items until they locate the target (see [92]). This pattern of subdividing visual–spatial stimuli into smaller units resulted in comparable saccadic patterns between participants regardless of their reading ability. Future research can further explore the visual pathway of saccadic patterns in simultaneous processing tasks and other reading-related tasks.

Finally, our study showed that the Grade 6 RD group had saccade measures similar to the Grade 3 RL-matched group, consistent with previous studies on consistent orthographies (e.g., [16,56]). Additionally, the Grade 6 children with RDs outperformed the younger control group in fixation measures, indicating that simultaneous processing skills are not a defining cause of reading difficulties in consistent orthographies (e.g., [24,57]). However, some studies have argued that this design is flawed for consistent orthographies. For instance, in a study with Greek-speaking children, Parrila and his colleagues [50] concluded that the reading-level match design was deemed “methodologically flawed as a tool for establishing causality in consistent orthographies” (p. 355). Similarly, in a study with Dutch children, van de Broeck and Geudens [104] concluded that the reading-level design is purely developmental, and any differences between RD and RL-matched groups can only be attributed to age-related factors. Therefore, more research is needed to determine whether the reading level-match design is appropriate for consistent orthographies.

The current findings have several educational and psychological implications, particularly for children with reading difficulties in orthographically consistent orthographies. First, the study highlights the need for reading research to employ advanced methodologies, such as eye-tracking and fixation-related potentials, to investigate the neuro-physiological basis of reading difficulties (e.g., [15,105]). Second, the findings suggest that digital tools designed for tailored intervention programs should aim to enhance eye movements in children with RDs through a practice that involves the simultaneous processing of verbal and spatial stimuli, especially in the early stages of reading [106,107]. Such remedial training has the potential to make important contributions to reading intervention research.

Finally, a few limitations need to be considered when interpreting the findings of our study. First, our research was conducted in Greek, so the results may only apply to languages with similar writing systems, such as German, Finnish, or Spanish. Second, while reading comprehension is the ultimate goal of reading (e.g., [108]), we did not measure it in this study (see also [109,110]). However, we made sure that the children who participated understood the instructions. Finally, we used one standard test to assess participants’ ability to process multiple elements simultaneously and form a cohesive whole (e.g., [111]). However, we recommend exploring other tests, such as Figure Memory or Matrices (DN:CAS, [82]), to further validate our findings about the connection between simultaneous processing and reading.

In conclusion, the current findings contribute to the existing research on using eye movement measures to differentiate between children with and without RDs in orthographically consistent languages. Further research is necessary to better understand the links between different cognitive and linguistic skills, reading difficulties and the development of reading skills. Using model-driven approaches with properly matched samples, developmental cohorts, carefully selected measures, and advanced methodologies, we can identify universal principles for reading skill development. This will help us to achieve our objective of improving reading skills in children with reading difficulties.

## Figures and Tables

**Table 1:** Demographic data for participating groups.

	RD 3rd Gr	CA/RL 3rd Gr	*x*^2^/*t*-Test	RD 6th Gr	CA 6th Gr	*x*^2^/*t*-Test
*Gender*			0.14			1.29
Males	8	9		11	8	
Females	7	6		4	7	
*Age in Years*			0.32			−1.87
Mean SD	8.30 (0.27)	8.36 (0.42)		11.01 (0.92)	11.47 (0.32)	

Note: RD: group with reading difficulties; CA: chronological-age matched group; RL: reading-level matched group; Gr: grade; all *ps* = ns.

**Table 2:** Descriptive statistics and F values for RD, CA, and RL groups for reading fluency and cognitive ability.

Variables	Groups								
	RD 3rd Gr		CA/RL 3rd Gr		RD 6th Gr		CA 6th Gr		*F*
	M	(SD)	M	(SD)	M	(SD)	M	(SD)	
*Reading Fluency*									
Word Reading	28.87	(6.35) ^2,3,4^	55.67	(8.16) ^4^	49.33	(7.22) ^4^	65.07	(7.03)	67.80 ***
Phonemic Decoding	18.73	(4.15) ^2,3,4^	32.20	(4.46) ^4^	29.27	(5.68) ^4^	39.53	(4.64)	49.13 ***
*Cognitive Ability*									
Vocabulary	20.73	(4.46) ^3,4^	21.33	(4.29) ^4^	25.47	(4.79)	27.33	(4.37)	7.64 ***
Nonverbal Matrices	12.87	(3.09)	12.27	(3.37) ^4^	16.13	(4.32)	16.13	(3.46)	5.00 **

Note: RD: group with reading difficulties; CA: chronological-age matched group; RL: reading-level matched group; superscript numbers indicate that group means differed significantly; Gr: grade; M = mean, SD = standard deviation; subscript letters indicate that group means differ significantly between each other; group comparisons are marked from left to right only: 2 = CA/RL 3rd Gr; 3 = RD 6th Gr; 4 = CA 6th Gr; ** *p* < 0.01; *** *p* < 0.001.

**Table 3:** Descriptive statistics and F values for RD, CA, and RL groups in the VSR task.

	RD 3rd Gr	CA/RL 3rd Gr	d ^1,2^	RD 6th Gr	d ^2,3^	CA 6th Gr	d ^3,4^	*F*
*Accuracy*								
Mean SD	14.40 (2.26)	17.00 (2.36) ^3,4^	1.13	17.93 (3.33)	0.32	17.40 (3.02)	0.17	4.79 **
*Speed*								
Mean SD	246.50 (19.32)	236.20 (11.53) ^3,4^	0.65	225.03 (10.77)	1.00	226.41 (15.39)	0.10	6.95 ***

Note: RD: group with reading difficulties; CA: chronological-age matched group; RL: reading-level matched group; Gr: grade; subscript letters indicate that group means differ significantly between each other; group comparisons are marked from left to right only: 1 = RD 3rd Gr; 2 = CA/RL 3rd Gr; 3 = RD 6th Gr; 4 = CA 6th Gr; ** *p* < 0.01; *** *p* < 0.001.

**Table 4:** Descriptive statistics and F values for RD, CA, and RL groups on the eye-tracking measures.

Variables	Groups											
	RD 3rd Gr		CA/RL 3rd Gr			RD 6th Gr			CA 6th Gr			*F*
	M	(SD)	M	(SD)	d ^1,2^	M	(SD)	d ^2,3^	M	(SD)	d ^3,4^	
Total number of fixations	26.76	(6.81) ^3,4^	21.44	(4.44)	0.93	18.87	(6.33)	0.47	19.76	(6.27)	0.14	5.15 **
Number of fixations—target	11.16	(3.50) ^3,4^	9.15	(2.27)	0.68	7.70	(2.98)	0.55	8.17	(2.48)	0.17	4.36 **
Number of fixations—distractor	15.82	(3.95) ^3,4^	12.29	(2.47)	1.07	10.31	(2.83)	0.75	11.59	(4.06)	0.37	6.30 ***
Total fixation duration	7.05	(2.80) ^3,4^	6.20	(1.43)	0.38	4.67	(1.81)	0.94	4.87	(1.52)	0.12	4.91 **
Fixation duration—target	3.08	(1.00) ^3,4^	2.68	(0.84)	0.43	2.11	(0.89)	0.66	2.09	(0.54)	0.02	4.94 **
Fixation duration—distractor	4.32	(1.34) ^3,4^	3.52	(0.76)	0.73	2.56	(1.01)	1.07	2.84	(1.04)	0.27	8.27 ***
Total number of saccades	29.03	(8.81) ^3,4^	23.54	(4.74)	0.78	20.99	(6.53)	0.45	21.76	(7.79)	0.11	3.88 *
Number of saccades—target	12.02	(3.77) ^3^	10.22	(2.18)	0.58	9.00	(2.85)	0.48	9.40	(2.48)	0.15	3.25 *
Number of saccades—distractor	17.00	(5.35) ^3,4^	13.31	(2.88)	0.86	12.33	(3.70)	0.30	12.53	(5.50)	0.04	3.52 *
Total saccade duration	1.40	(0.48)	1.14	(0.34)	0.63	1.14	(0.42)	0.00	1.18	(0.56)	0.08	1.07
Saccade duration—target	0.58	(0.24)	0.49	(0.20)	0.41	0.45	(0.19)	0.21	0.47	(0.22)	0.09	1.02
Saccade duration—distractor	0.82	(0.26)	0.65	(0.16)	0.79	0.57	(0.24)	0.39	0.71	(0.37)	0.45	1.15

Note: RD: group with reading difficulties; CA: chronological-age matched group; RL: reading-level matched group; Gr: grade; M = mean, SD = standard deviation; subscript letters indicate that group means differ significantly between each other; group comparisons are marked from left to right only: 1 = RD 3rd Gr; 2 = CA/RL 3rd Gr; 3 = RD 6th Gr; 4 = CA 6th Gr; * *p* < 0.05; ** *p* < 0.01; *** *p* < 0.001.

## Data Availability

Further data analysis is currently in progress. Data sharing is not applicable to this article.
